# Psychotropic drug use and mortality in old people with dementia: investigating sex differences

**DOI:** 10.1186/s40360-017-0142-9

**Published:** 2017-05-25

**Authors:** Jon Brännström, Gustaf Boström, Erik Rosendahl, Peter Nordström, Håkan Littbrand, Hugo Lövheim, Yngve Gustafson

**Affiliations:** 1 Department of Community Medicine and Rehabilitation, Geriatric Medicine, Umeå University, Umeå, Sweden; 2 Department of Community Medicine and Rehabilitation, Physiotherapy, Umeå University, Umeå, Sweden

**Keywords:** Alzheimer’s disease, Antidepressants, Antipsychotics, Benzodiazepines, Cohort study, Dementia, Gender, Mortality, Old age, Psychotropic drugs, Vascular dementia

## Abstract

**Background:**

Psychotropic drugs are common among old people with dementia, and have been associated with increased mortality. Previous studies have not investigated sex differences in this risk. This study was conducted to analyse associations between the use of antipsychotics, antidepressants, and benzodiazepines and 2-year mortality in old people with dementia, and to investigate sex differences therein.

**Methods:**

In total, 1037 participants (74% women; mean age, 89 years) with dementia were included from four cohort studies and followed for 2 years. Data were collected through home visits and medical records. Cox proportional hazard regression models were used to analyse associations between ongoing baseline drug use and mortality. Multiple possible confounders were evaluated and adjusted for.

**Results:**

In fully adjusted models including data from the whole population, no association between baseline psychotropic drug use and increased 2-year mortality was seen. Significant sex differences were found in mortality associated with antidepressant use, which was protective in men, but not in women (hazard ratio [HR] 0.61, 95% confidence interval [CI] 0.40–0.92 and HR 1.09, 95% CI 0.87–1.38, respectively). The interaction term for sex was significant in analyses of benzodiazepine use, with a higher mortality risk among men than among women.

**Conclusions:**

Among old people with dementia, ongoing psychotropic drug use at baseline was not associated with increased mortality in analyses adjusted for multiple confounders. Sex differences in mortality risk associated with antidepressant and benzodiazepine use were seen, highlighting the need for further investigation of the impact of sex.

**Electronic supplementary material:**

The online version of this article (doi:10.1186/s40360-017-0142-9) contains supplementary material, which is available to authorized users.

## Background

### Introduction

Psychotropic drug use is common among old people, especially those suffering from dementia. In Sweden and Finland, every second old person with dementia uses psychotropic drugs [1, 2]. The prevalence is even higher among nursing home residents with dementia, of whom four of five are prescribed psychotropic drugs [3, 4]. In comparison, one third of old people without dementia are psychotropic drug users [1, 2]. One plausible explanation for this high rate of prescription is that these drugs are used to treat behavioural and psychological symptoms of dementia (BPSD), although many elderly may lack diagnosis [5, 6]. BPSD, include, but are not limited to, hallucinations, anxiety, depression, agitation, and aggression. Current guidelines propose psychosocial and non-pharmacological interventions to reduce BPSD, but also refer to psychotropic drug administration, generally as a second line of defence [7–10], even though their clinical effectiveness has been questioned [11–13], and they are all associated with significant side effects, some of which are sex-specific [14–20].

In a meta-analysis of randomised control trials published 2005, antipsychotic drug use increased mortality with about 50% [21]. In addition, several more recent studies have demonstrated increased mortality associated with the use of antipsychotics [22–27]. Also antidepressants have been associated with increased mortality in old people in general [16, 28–30], and in one study of old men with dementia [26]. Increased mortality associated with antipsychotic and antidepressant drug use has been shown to be most pronounced during the first period after treatment initiation [16, 22, 24]. In contrast, benzodiazepines have not been associated with mortality in old people [31–34].

Most of the observational studies conducted to date have been registry studies [23, 24, 26, 28, 32, 35], which provide insufficient control for factors associated with drug use, and thus present difficulties with limitation of the effects of possible confounding by indication. Other than for antipsychotics, knowledge of the associations between psychotropic drug use and mortality, specifically in people with dementia, is lacking. In addition, few studies have investigated possible sex differences in drug-related mortality in old people with dementia.

### Aims

The aims of this study were to explore the association between psychotropic drug use and 2-year mortality in old people with dementia, and to investigate possible sex differences in this outcome.

## Methods

### Design and settings

To achieve a sufficiently large sample, enabling control for several possible indicators of health and mortality, data from four studies were merged: Umeå 85+/Gerontological Regional Database (GERDA, 2000–2012); Frail Older People–Activity and Nutrition (FOPANU, 2002); Residential Care Facilities–Mobility, Activity and Nutrition (REMANU, 2004); and Umeå Dementia and Exercise (UMDEX, 2012).

The GERDA epidemiological cohort study was initiated in 2000 to investigate factors impacting the general health and well-being of very old people in one urban municipality and five rural municipalities in the county of Västerbotten, northern Sweden. Half of the residents aged 85 years, all 90-year-olds, and all of those aged ≥ 95 years were invited to participate. Age was the only inclusion criterion and no exclusion criterion was used. Different levels of participation were possible; for example, participants could allow access to medical charts and permit interviews with caregivers and next of kin, but decline home visitation. The initial data collection phase was completed in 2002. Between 2005 and 2007, a second round of data collection was performed in the same municipalities, as well as in two municipalities in the Finnish county of Pohjanmaa. Between 2010 and 2012, a third round of data collection was performed in the same areas, with the addition of two Finnish municipalities. This study has been described in greater detail elsewhere [36]. Seventy-one percent of participants in the current study were recruited from the GERDA study.

Baseline data were obtained from FOPANU and UMDEX, randomised controlled trials with exercise interventions, and REMANU, an observational study with similar inclusion criteria. They were all performed in the urban municipality of Umeå. The inclusion criteria for these studies were: residence in a nursing home, age ≥ 65 years, dependency in personal activities of daily living (ADL), ability to rise from a chair with armrests with help from no more than one person, Mini-Mental State Examination (MMSE) score ≥ 10, and approval from the resident’s physician. The UMDEX study included only people with dementia. These studies have been described in greater detail elsewhere [37–39].

Common traits of all four studies were that data collection was conducted by trained investigators (physicians, nurses, physiotherapists, and medical students), mainly during home visits, through structured interviews and various measurements and assessments. Additional data were collected in interviews with caregivers and/or next of kin, as well as from medical charts.

### Participants

**Fig. 1:**
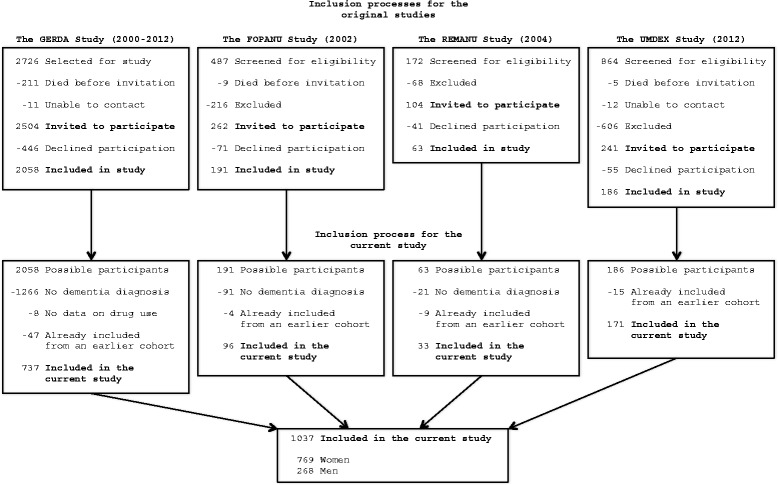
Flow chart of the inclusion process for this study

Included in the current study were all participants with a diagnosis of dementia who had provided information on their use of prescription drugs. For people participating in more than one of the four studies, only data from the first study were included. Of the 2498 participants in the four original studies, 1037 met our inclusion criteria. The lack of a diagnosis of dementia led to the exclusion of 1378 persons, data on prescribed drugs were missing for 8 persons, and data from 75 persons were excluded because they had already been included from another of the four studies. The procedure for study inclusion is illustrated in Fig. 1.

### Measurements and data

Cognitive function was assessed using the MMSE (range, 0–30; higher scores indicate higher function) [40], screening for depressive symptoms was conducted using the 15-item version of the Geriatric Depression Scale (GDS-15; range, 0–15; higher scores indicate more depressive symptoms) [41], morale was assessed using the Philadelphia Geriatric Center Morale Scale (range, 0–17; higher scores indicate higher morale) [42], nutritional status was evaluated using the Mini Nutritional Assessment (MNA; range, 0–30; higher scores indicate lower risk of malnutrition) [43], body mass index (BMI; weight [in kilograms]/height [in meters]^2^) was calculated, and dependency in ADL was measured with the Barthel ADL index (range, 0–20; higher scores indicate greater independence) [44]. Information regarding medical diagnoses and symptoms was collected from medical charts and in structured interviews, and was classified according to the International Classification of Diseases, 10th Revision [45]. Diagnoses of dementia, delirium, and depressive disorders were established in accordance with the Diagnostic and Statistical Manual of Mental Disorders, 4th Edition [46]. A specialist in geriatric medicine reviewed all diagnoses, using information from medical charts and from assessments conducted during data collection. Data on medications were obtained from lists of prescribed drugs, as well as by asking the participants and their caregivers about over-the-counter drug use and actual drug use. Drugs were grouped according to the Anatomical Therapeutic Chemical (ATC) index. The definition of antipsychotic drugs used in the current study encompassed drugs with close kinship to conventional antipsychotics, resulting in the inclusion of N05A (but not N05AN01 [lithium]), N05BB01, N05CM06, and R06AD. Antidepressant drugs were defined as all drugs within ATC code N06A. The analyses of benzodiazepines also included benzodiazepine-like Z-drugs, hereafter referred to collectively as benzodiazepines; hence, this category included all drugs classified as N05BA, N05CD, N05CF, and N03AE. Scheduled medications, as well as those used *pro re nata* (as needed), were included, provided that participants actually used them. Dates of death during the 2-year follow-up period were collected from the population registers provided by the Swedish Tax Agency and the Population Register Centre in Finland. Sex was classified dichotomously.

### Statistical analysis

Associations between drug use and mortality were analysed with Cox proportional hazards regression models. The selection of possibly confounding variables for the multivariate models was made according to findings reported in the scientific literature and the authors’ clinical experience. Fifty variables were evaluated by investigating differences between users and non-users of antipsychotics, antidepressants, and benzodiazepines, respectively, using the chi-squared test and Student’s *t*-test, as well as by analysing associations between the selected variables and mortality using univariate Cox models. Variables with *p* values ≤ 0.15 for group differences and associations with mortality were included in the multivariate Cox regression models. This procedure was conducted for the full sample and separately for men and women to detect sex-specific confounders. Age and sex were included in the models, regardless of their associations with the outcome or indicator, as were GDS-15 scores for the analyses of antidepressants. Some variables were excluded due to singularity or multicollinearity (Pearson’s or Spearman’s correlation coefficient, *r* ≥ 0.6). When the selection process resulted in the inclusion of more variables than one-tenth the number of events, stepwise backward removal was conducted. A list of all investigated and included/excluded confounders is provided in Additional file 1. Sensitivity analyses were performed, where all variables that might be considered mediators, rather than confounders, were removed. Sex-specific analyses including the variables selected for all participants were also performed. These analyses are presented in Additional file 1.

Following the selection of confounders, proportionality of hazards was investigated through Schoenfeld residuals. When time-dependent covariates were identified, extended Cox regression models were used to evaluate associations with mortality. The associations between benzodiazepines and 2-year mortality were found to be time dependent for men and for the full sample, which made the Cox regression model unsuitable for these analyses. Hence, all analyses of benzodiazepines were split in two, with separate analyses of the first and second years following baseline, after the investigation of Kaplan–Meier curves and Schoenfeld residuals for these time periods.

Interaction terms were created to further investigate possible sex differences in mortality by multiplying the sex variable by each dichotomous variable indicating use of the studied drugs (sex * drug). Interaction terms consisting of MMSE score and drug use were also used to investigate whether the associations between psychotropic drug use and mortality were dependent on cognitive level (mmse * drug). These interaction terms were then entered in the multivariate Cox regression models. As the interaction term for MMSE score was non-significant in all models (data not shown), the impact of level of cognitive functioning on mortality was not investigated further.

Some variables had missing data, which was considered to constitute bias because it was associated with higher morbidity and lower scores on several scales. Hence, multiple imputation (20 sets) was conducted to fill the gaps for the variables of GDS-15 score, MMSE score, BMI, Barthel ADL index, and myocardial infarction >1 year before baseline. The proportions of missing values for these variables ranged from 6.5 to 35.1% (Table 1). Variables used as predictors in the imputation model were those included in the Cox regression models, those correlating with the variables to be imputed (*r* ≥ 0.3), and those correlating with the presence of missing values for those variables (Additional file 1) [47]. No restriction was set for the imputed values.

**Table 1:** Participant characteristics

	Total	Men	Women	Alive	Deceased
	All				
	*n = 1037*	*n = 268*	*n = 769*	*n = 549*	*n = 488*
Age	89.4 ± 6.2	88.0 ± 5.9	89.9 ± 6.3*	87.9 ± 6.4	91.0 ± 5.5*
Sex, female	769 (74)			412 (75)	357 (73)
Deceased ≤ 2 years	488 (47)	131 (49)	357 (46)		
Medical conditions and diagnoses					
Alzheimer's disease	536 (52)	135 (50)	401 (52)	309 (56)	227 (47)*
Vascular dementia	398 (38)	121 (45)	277 (36)*	196 (36)	202 (41)
Other dementia	137 (13)	31 (12)	106 (14)	68 (12)	69 (14)
Non-specified dementia	62 (6)	11 (4)	51 (7)	30 (5)	32 (7)
Delirium, last month	494 (48)	126 (47)	368 (48)	221 (40)	273 (56)*
Depressive disorder	525 (51)	129 (48)	396 (51)	276 (50)	249 (51)
Angina pectoris	370 (36)	104 (39)	266 (35)	171 (31)	199 (41)*
Atrial fibrillation	213 (21)	66 (25)	147 (19)	98 (18)	115 (24)*
Congestive heart failure	359 (35)	87 (32)	272 (35)	150 (27)	209 (43)*
Diabetes mellitus	165 (16)	55 (21)	110 (14)*	86 (16)	79 (16)
Hip fracture, ever	187 (18)	32 (12)	155 (20)*	96 (17)	91 (19)
Malignant disease, ever	196 (19)	71 (26)	125 (16)*	100 (18)	96 (20)
Myocardial infarction, ever (12.2% imputed)	198 (19)	61 (23)	137 (18)	86 (16)	112 (23)*
Stroke, ever	257 (25)	78 (29)	179 (23)	124 (23)	133 (27)
Prescribed drugs					
Antipsychotics	230 (22)	54 (20)	176 (23)	113 (21)	117 (24)
Antidepressants	388 (37)	94 (35)	294 (38)	203 (37)	185 (38)
Bensodiazepines	404 (39)	87 (32)	317 (41)*	205 (37)	199 (41)
Analgesics	691 (67)	154 (57)	537 (70)*	332 (60)	359 (74)*
Antiepileptics	47 (5)	13 (5)	34 (4)	27 (5)	20 (4)
Cholinesterase inhibitors	163 (16)	52 (19)	111 (14)	100 (18)	63 (13)*
Memantine	38 (4)	11 (4)	27 (4)	22 (4)	16 (3)
Number of prescribed drugs	7.7 ± 3.8	7.2 ± 3.9	7.9 ± 3.8*	7.1 ± 3.6	8.4 ± 4.0*
Scales and measurements					
Barthel ADL index (6.5% imputed)	12.0 ± 6.1	13.2 ± 5.9	11.6 ± 6.2*	13.8 ± 5.5	9.9 ± 6.2*
BMI (14.9% imputed)	25.2 ± 4.9	25.4 ± 4.3	25.1 ± 5.1	26.0 ± 4.9	24.3 ± 4.8*
GDS-15 (35.1% imputed)	4.2 ± 3.0	4.1 ± 3.1	4.2 ± 2.9	4.0 ± 2.9	4.4 ± 3.1*
MMSE (14.2% imputed)	13.8 ± 6.7	15.3 ± 6.0	13.2 ± 6.8*	15.5 ± 6.1	11.8 ± 6.8*

Notes: Results are presented as mean ± standard deviation or as number (%). Group differences marked with * are statistically significant (*p* < 0.05). *ADL* Activities of Daily Living, *BMI* Body Mass Index, *GDS-15* Geriatric Depression Scale, 15-item version, *MMSE* Mini Mental State Examination

All mortality analyses were conducted on the original data set and the data set completed through imputation. The two sets of results for the main outcomes were close to identical. The results presented in the present paper, come from the imputed data set. All statistical analyses and calculations were performed using IBM SPSS Statistics software (version 23; IBM Corporation, Armonk, NY, USA).

## Results

### Baseline data

The study population comprised 1037 people with dementia aged ≥ 65 years (mean age, 89.4 ± 6.2 years); 74.2% of participants were women and 47.1% died during the 2-year follow-up period. Alzheimer’s disease was the most common type of dementia, affecting 51.7% of the population, followed by vascular dementia, which had a prevalence of 38.4%. The most common co-morbidities were depressive disorder (50.6%), recent delirium (47.6%), and angina pectoris (35.7%).

The female participants in this study were slightly older, had lower MMSE scores and Barthel ADL index scores and used more drugs, specifically benzodiazepines and analgesics, than did their male counterparts. Comparison of participants who were alive with those who died during the 2-year follow-up period revealed larger differences. A more detailed description of the participants can be found in Table 1.

Psychotropic drugs prescribed at the time of data collection are listed in Table 2. Antidepressants were used by 37.4% of the participants, antipsychotics by 22.2%, and benzodiazepines were used by 39.0% of participants; 63.5% of participants used at least one drug from these three groups. A smaller proportion (18.3%) had ongoing treatment with anti-dementia drugs. Sex differences were seen in the use of several types of benzodiazepines.

**Table 2:** Psychotropic drug use at baseline

ATC-code	Drug group	Total	Men	Women
		*n = 1037*	*n = 268*	*n = 769*
N05A-(N05AN01) + N05BB01 + N05CM06 + R06AD	Antipsychotics and related (incl. PRN)	230 (22)	54 (20)	176 (23)
N05A-(N05AN01) + N05BB01 + N05CM06 + R06AD	Antipsychotics and related (excl. PRN)	219 (21)	51 (19)	168 (22)
N05A-(N05AN01)	Antipsychotics (incl. PRN)	175 (17)	37 (14)	138 (18)
N05A-(N05AN01)	Antipsychotics (excl. PRN)	168 (16)	36 (13)	132 (17)
N05BB01 + N05CM06 + R06AD	Drugs related to antipsychotics (incl. PRN)	72 (7)	20 (7)	52 (7)
N05BB01 + N05CM06 + R06AD	Drugs related to antipsychotics (excl. PRN)	67 (6)	18 (7)	49 (6)
	Typical antipsychotics (incl. PRN)	53 (5)	12 (4)	41 (5)
	Typical antipsychotics (excl. PRN)	45 (4)	9 (3)	36 (5)
	Atypical antipsychotics (incl. PRN)	135 (13)	30 (11)	105 (14)
	Atypical antipsychotics (excl. PRN)	132 (13)	30 (11)	102 (13)
N06A	Antidepressants (No PRN use)	388 (37)	94 (35)	294 (38)
N06AA	Tricyclic antidepressants	14 (1)	3 (1)	11 (1)
N06AB	SSRIs	299 (29)	81 (30)	218 (28)
N06AG	MAO-inhibitors	1 (0)	0 (0)	1 (0)
N06AX	Other antidepressants	115 (11)	20 (7)	95 (12)*
N06AX03 + N06AX11	Tetracyclic antidepressants	89 (9)	17 (6)	72 (9)
N06AX16 + N06AX21	SNRIs	28 (3)	3 (1)	25 (3)
N05BA + N05CD + N05CF + N03AE	BZD and Z-drugs (including PRN)	404 (39)	87 (32)	317 (41)*
N05BA + N05CD + N05CF + N03AE	BZD and Z-drugs (excluding PRN)	304 (29)	60 (22)	244 (32)*
N05BA	Anxiolytics (incl. PRN)	161 (16)	26 (10)	135 (18)*
N05BA	Anxiolytics (excl. PRN)	99 (10)	14 (5)	85 (11)*
N05CD + N05CF	Hypnotics, BZD and Z-drugs (incl. PRN)	312 (30)	69 (26)	243 (32)
N05CD + N05CF	Hypnotics, BZD and Z-drugs (excl. PRN)	250 (24)	52 (19)	198 (26)*
N05CD	Hypnotics, BZD (incl. PRN)	74 (7)	20 (7)	54 (7)
N05CD	Hypnotics, BZD (excl. PRN)	64 (6)	16 (6)	48 (6)
N05CF	Hypnotics, Z-drugs (incl. PRN)	239 (23)	50 (19)	189 (25)*
N05CF	Hypnotics, Z-drugs (excl. PRN)	186 (18)	36 (13)	150 (20)*
N03AE	BZD derivatives (incl. PRN)	6 (1)	2 (1)	4 (1)
N03AE	BZD derivatives (excl. PRN)	5 (0)	1 (0)	4 (1)
	Any psychotropic drug	659 (64)	159 (59)	500 (65)
	BZD and antidepressants	186 (18)	38 (14)	148 (19)
	BZD and antipsychotics	129 (12)	25 (9)	104 (14)
	Antidepressants and antipsychotics	113 (11)	24 (9)	89 (12)
	BZD, antidepressants and antipsychotics	65 (6)	11 (4)	54 (7)

Notes: Results are presented as number (%). Significant sex differences are marked with * (*p* < 0.05). The results of combinations of psychotropic drugs include drugs related to antipsychotics and benzodiazepines. *PRN* Pro Re Nata (when needed), *SSRIs* Selective Serotonin Reuptake Inhibitors, *MAO* Monoamine Oxidase, *SNRIs* Serotonin-Norepinephrine Reuptake Inhibitors, *BZD* Benzodiazepines

**Fig. 2:**
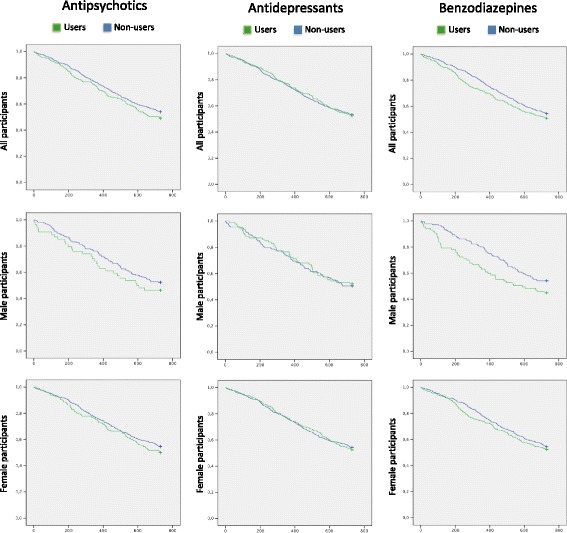
Unadjusted Kaplan–Meier survival curves. Notes: *Green lines* signify people with ongoing drug treatment at baseline, whereas *blue lines* signify non-users at baseline. Time, in days, is placed on the *x-axis* and cumulative survival on the *y-axis*. The analyses of antipsychotics and benzodiazepines include related drugs, as described in methods

Fig. 2 shows the unadjusted Kaplan–Meier survival curves. Results from the Cox proportional hazard regression models are presented in Table 3.

**Table 3:** Cox proportional hazards regression models

	Model 1		Model 2		Interaction Term
	HR	95% CI	HR	95% CI	
Antipsychotics (2 year mortality)					sex*antipsychotics
All participants	1.16	0.94–1.43	0.91	0.73–1.14	*p* = 0.962
Male participants	1.24	0.82–1.87	0.79	0.51–1.24	
Female participants	1.15	0.90–1.46	0.90	0.70–1.15	
Antidepressants (2 year mortality)					sex*antidepressants
All participants	1.01	0.84–1.21	0.96	0.78–1.17	*p* = 0.047
Male participants	0.96	0.67–1.37	0.61*	0.40–0.92	
Female participants	1.03	0.84–1.28	1.09	0.87–1.38	
Benzodiazepines (first-year mortality)					sex*benzodiazepines
All participants	1.38*	1.08–1.77	1.13	0.86–1.47	*p* = 0.029
Male participants	2.07*	1.29–3.32	1.37	0.77–2.45	
Female participants	1.21	0.91–1.62	0.96	0.71–1.31	
Benzodiazepines (second-year mortality)					sex*benzodiazepines
All participants	0.95	0.73–1.24	0.72*	0.54–0.96	*p* = 0.964
Male participants	0.90	0.51–1.59	0.81	0.45–1.45	
Female participants	0.98	0.72–1.33	0.73	0.53–1.02	

Notes: Analyses of antipsychotics and benzodiazepines include related drugs, as described in methods. Hazard ratios marked with * are statistically significant (*p* < 0.05). *HR* Hazard ratio. *CI* Confidence interval. Model 1 shows the unadjusted, univariate associations between drug use and mortality. Model 2 includes all available confounders, selected through the process described in the statistics section. Interaction Term shows the p-values for the respective interaction terms applied to Model 2

### Antipsychotics

No association between baseline use of antipsychotics and mortality was observed in any analysis. The interaction term for sex was not significant. No significant difference in association with mortality was seen between users of typical and atypical antipsychotics, in the entire sample or among men or women (data not shown).

### Antidepressants

No association between antidepressant use and mortality was seen in the univariate analysis or the fully adjusted model for the entire sample. The interaction term for sex was significant (*p* = 0.047). Antidepressant use was associated significantly with lower 2-year mortality in men (HR, 0.61; 95% confidence interval [CI], 0.40–0.92), but not in women.

### Benzodiazepines, including Z-drugs

The use of benzodiazepines at baseline was associated with increased mortality during the first year thereafter in the unadjusted model (HR, 1.38; 95% CI, 1.08–1.77), but not in the fully adjusted model. The unadjusted model showed no association with mortality risk during the second year, but the fully adjusted model revealed an association between benzodiazepine use and decreased mortality during this period (HR, 0.72; 95% CI, 0.54–0.96). The interaction term for sex was significant for the first year (*p* = 0.029), but not the second year (*p* = 0.964), after baseline. First-year mortality associated with benzodiazepine use was elevated in men in the univariate analysis (HR, 2.07; 95% CI, 1.29–3.32), but not in the fully adjusted model. No significant association was seen for men during the second year or for women in any model.

## Discussion

In this study of old people with dementia, no association was found between baseline psychotropic drug use and increased 2-year mortality in fully adjusted models including all participants. This is in contrast to recently published registry studies [24, 26, 28, 35]. Significant sex differences were found in mortality associated with the use of antidepressant drugs and benzodiazepines.

### Antipsychotics

The finding that antipsychotic use was not associated with an elevated mortality risk in either analysis was not in line with previous research, most of which has shown an increased risk of mortality [21–27]. A liberal definition of this drug group was used, but the results of subgroup analyses of typical and atypical antipsychotics remained consistent. In other studies, the increased risk of mortality associated with the use of antipsychotics has been shown to be more pronounced in approximately the first month after treatment initiation [22, 24], although a randomised placebo-controlled withdrawal trial also showed increased long-term mortality in users of antipsychotics [27]. As the baseline date was not set in relation to the first ingested dose of a psychotropic drug in the current study, we did not expect to observe this effect of higher initial mortality; that period had probably already passed for the majority of the participants on psychotropic medication. Conversely, all non-users at baseline who started using psychotropic drugs during the 2-year follow-up period would have been subjected to this higher risk of fatal side effects, thereby possibly increasing mortality rates in the non-user groups. The side effects of antipsychotics, fatal and otherwise, may be more likely to affect the frailest old people with dementia, which may result in antipsychotic treatment termination among those not tolerating treatment. In our study, this factor could have resulted in a healthy worker effect – a sampling bias in which those defined as users of antipsychotics constituted a selected group of more resilient individuals.

### Antidepressants

The findings of this study with regard to antidepressants contrast with those of recent registry studies of old people [16, 26, 28, 35], two of which specifically investigated people with dementia and all of which demonstrated increased mortality associated with antidepressant drug use. This difference could be explained in part by the baseline date, which was not set according to the prescription date, in the present study. Just as with antipsychotic use, higher initial mortality has been shown to be associated with antidepressant use [16]. Another explanatory factor could be the ability to adjust extensively for confounders in the present study, as the participants were recruited from studies in which they had been assessed thoroughly. Treatment with antidepressant drugs was associated with better survival for men, but not for women. These results contrast with findings reported by Ryan et al. [29], who observed an association between antidepressant use and increased mortality in old men. The characteristics of participants in the two studies, however, differ substantially, as Ryan et al. [29] did not examine people with dementia. Most antidepressant drugs have the potential to alter the conductive properties of the heart, giving rise to QT prolongation, an effect that is more pronounced in women than in men [48]. This effect can lead to potentially fatal arrhythmia (Torsade de Pointes) [49]. Other antidepressants (N06AX), including mirtazapine and venlafaxine, which were used more frequently by female participants in the current study, are among those associated most strongly with increased mortality in previous studies [16, 28]. These two conditions, if also true in people with dementia, may lead to more adverse effects of antidepressants in women, nullifying the protective effects observed in men.

### Benzodiazepines, including Z-drugs

A time-dependent association between mortality and the use of benzodiazepines was observed in the present study, resulting in separate analyses of the first and second years after baseline. A tendency toward increased mortality among benzodiazepine users during the first year was observed, but the results were significant only in univariate analyses. Sex differences were seen in mortality, which was elevated in men, but this result was significant only in the unadjusted analysis. Nevertheless, the interaction term for sex was significant and sex differences in mortality could possibly be moderated by prolonged sleep apnea events induced by benzodiazepines [50], which has been shown to increase the risk for ischemic stroke in men, but not in women [51].

The fully adjusted models showed an association between baseline use and lower second-year mortality. As information on how many benzodiazepine users became non-users, and vice versa, was not available, we are quite uncertain of whether these results would be reproduced in a study with a more controlled design. A recent observational study of people with dementia showed that about half of baseline benzodiazepine users were still using the same medication 6 months later [3]. In a registry study, Jennum et al. [35] found no association between benzodiazepine use and mortality in people with dementia, but they did not investigate sex differences.

### Strengths and weaknesses

The majority (71%) of participants in this study were included from a cohort with no exclusion criterion, and the remaining participants were included from studies that excluded those with severe dementia (<10 on MMSE), but otherwise had few exclusion criteria. People with all types and severities of dementia were included, with the exception of home-dwelling people aged 65–85 years, as the studies including the younger old were conducted at nursing homes. Hence, the study sample should be quite representative of old people with dementia. Strong data on the clinical characteristics of the participants was available, trained investigators assessed most participants in person and reviewed all medical charts, and a specialist in geriatric medicine verified all diagnoses. In the current study, all data except dates of death were collected only at baseline. Therefore, it is difficult to distinguish between confounding factors and factors that are mediators of the associations between drug use and mortality. However, sensitivity analyses, with suspected mediators removed, did not differ from our main results (Additional file 1). Data on drugs were collected from lists of prescriptions and verified by participants and/or their caregivers, resulting in good knowledge of actual drug use. The ability to analyse specific medications or different doses, rather than groups of drugs, however, was limited by the number of participants.

In our opinion, the most significant limitation of this study is that we were not able to include the timeline of drug use in the analyses. The dichotomisation of participants as users and non-users of the respective drugs was performed according to baseline status. We lacked data on the length of drug use before baseline and whether use continued during the follow-up period. We also did not know how many non-users at baseline became users during the follow-up period. However, previous studies in the same geographic regions have shown that the use of psychotropic drugs, especially antidepressants and antipsychotic, among old people with dementia is typically long term [3, 52].

### Implications

Although no association between ongoing psychotropic drug use at baseline and elevated 2-year mortality was found, the clinical implications of the current study should not be interpreted as if there is no increased risk of death associated with the initiation of treatment with psychotropic drugs in old people with dementia, as previous research has shown increased mortality soon after initiation. One implication to be considered by researchers is that the HRs were almost invariably reduced in the fully adjusted models, compared with the less controlled models. This finding implies that thorough consideration of confounders is necessary when examining the impact of psychotropic drug use on mortality in old people with dementia. Our results also highlight the importance of sex-specific analyses – at the very least, presentation of sex-disaggregated results – as confounders, as well as mortality, differed between men and women in the current study.

## Conclusions

In this study of old people with dementia, we found less pronounced associations between psychotropic drug use and mortality than demonstrated in previous studies. Our data were taken from four cohort studies in which the participants were assessed thoroughly, in contrast to previous research, much of which has been in the form of registry studies. This distinction highlights the importance of extensive investigation of confounders. We found sex differences in mortality associated with the use of antidepressants and benzodiazepines, highlighting the need to account sufficiently for sex in future research.

## Additional file

Additional file 1: Statistical procedures. (DOCX 90 kb)

## Abbreviations

ADL: Activities of daily living
ATC: Anatomical Therapeutic Chemical index
BMI: Body mass index
BPSD: Behavioural and psychological symptoms of dementia
CI: Confidence interval
FOPANU: Frail Older People–Activity and Nutrition
GDS-15: 15-item version of the Geriatric Depression Scale
GERDA: Umeå 85+/Gerontological Regional Database
HR: Hazard ratio
MMSE: Mini-Mental State Examination
MNA: Mini Nutritional Assessment
OR: Odds ratio
REMANU: Residential Care Facilities–Mobility, Activity and Nutrition
UMDEX: Umeå Dementia and Exercise

## References

[1] Giron MS, Forsell Y, Bernsten C, Thorslund M, Winblad B, Fastbom J (2001). Psychotropic drug use in elderly people with and without dementia. Int J Geriatr Psychiatry.

[2] Taipale H, Koponen M, Tanskanen A, Tolppanen AM, Tiihonen J, Hartikainen S (2014). High prevalence of psychotropic drug use among persons with and without Alzheimer's disease in Finnish nationwide cohort. Eur Neuropsychopharmacol.

[3] Gustafsson M, Karlsson S, Gustafson Y, Lövheim H (2013). Psychotropic drug use among people with dementia--a six-month follow-up study. BMC Pharmacol Toxicol.

[4] Hosia-Randell H, Pitkala K (2005). Use of psychotropic drugs in elderly nursing home residents with and without dementia in Helsinki, Finland. Drugs Aging.

[5] Selbaek G, Kirkevold O, Engedal K (2007). The prevalence of psychiatric symptoms and behavioural disturbances and the use of psychotropic drugs in Norwegian nursing homes. Int J Geriatr Psychiatry.

[6] Gustafsson M, Sandman PO, Karlsson S, Gustafson Y, Lövheim H (2013). Association between behavioral and psychological symptoms and psychotropic drug use among old people with cognitive impairment living in geriatric care settings. Int Psychogeriatr.

[7] Ballard C, Corbett A (2013). Agitation and aggression in people with Alzheimer's disease. Cur Opin Psychiatry.

[8] Livingston G, Kelly L, Lewis-Holmes E, Baio G, Morris S, Patel N, Omar RZ, Katona C, Cooper C (2014). A systematic review of the clinical effectiveness and cost-effectiveness of sensory, psychological and behavioural interventions for managing agitation in older adults with dementia. Health Technol Assess.

[9] Press DA, M. Management of neuropsychiatric symptoms of dementia: Wolters Kluwer; 2015 Available from: http://www.uptodate.com/contents/management-of-neuropsychiatric-symptoms-of-dementia. Accessed 15 Jan 2016.

[10] Läkemedelsverket – the Medical Products Agency in Sweden. Treatment of Behavioural and Psychological Symptoms of Dementia – BPSD – new guidelines. (Läkemedelsbehandling och bemötande vid Beteendemässiga och Psykiska Symtom vid Demenssjukdom - BPSD - ny rekommendation. Information från Läkemedelsverket.) 2008;19(5).

[11] Schneider LS, Tariot PN, Dagerman KS, Davis SM, Hsiao JK, Ismail MS, Lebowitz BD, Lyketsos CG, Ryan JM, Stroup TS, Sultzer DL, Weintraub D, Lieberman JA, Catie-Ad Study Group (2006). Effectiveness of atypical antipsychotic drugs in patients with Alzheimer's disease. N Engl J Med.

[12] Banerjee S, Hellier J, Dewey M, Romeo R, Ballard C, Baldwin R, Bentham P, Fox C, Holmes C, Katona C, Knapp M, Lawton C, Lindesay J, Livingston G, McCrae N, Moniz-Cook E, Murray J, Nurock S, Orrell M, O’Bries J, Poppe M, Thomas A, Walwyn R, Wilson K, Burns A (2011). Sertraline or mirtazapine for depression in dementia (HTA-SADD): a randomised, multicentre, double-blind, placebo-controlled trial. Lancet.

[13] Wilkinson P, Izmeth Z (2012). Continuation and maintenance treatments for depression in older people. Cochrane Database Syst Rev.

[14] Holt S, Schmiedl S, Thurmann PA (2010). Potentially inappropriate medications in the elderly: the PRISCUS list. Dtsch Arztebl Int.

[15] Laroche ML, Perault-Pochat MC, Ingrand I, Merle L, Kreft-Jais C, Castot-Villepelet A, Durrieu G, Gras V, Guy C, Jean-Pastor MJ, Jonville-Bera AP, Merlet-Chicoine I, Miremont-Salame G, Nourhashemi F, Charmes JP, French Ctr Pharmacovigilance (2013). Adverse drug reactions in patients with Alzheimer's disease and related dementia in France: a national multicentre cross-sectional study. Pharmacoepidemiol Drug Saf.

[16] Coupland C, Dhiman P, Morriss R, Arthur A, Barton G, Hippisley-Cox J (2011). Antidepressant use and risk of adverse outcomes in older people: population based cohort study. BMJ.

[17] Moore AR, O'Keeffe ST (1999). Drug-induced cognitive impairment in the elderly. Drugs Aging.

[18] van Strien AM, Koek HL, van Marum RJ, Emmelot-Vonk MH (2013). Psychotropic medications, including short acting benzodiazepines, strongly increase the frequency of falls in elderly. Maturitas.

[19] Hajjar ER, Hanlon JT, Artz MB, Lindblad CI, Pieper CF, Sloane RJ, Ruby CM, Schmader KE (2003). Adverse drug reaction risk factors in older outpatients. Am J Geriatr Pharmacother.

[20] Haack S, Seeringer A, Thurmann PA, Becker T, Kirchheiner J (2009). Sex-specific differences in side effects of psychotropic drugs: genes or gender?. Pharmacogenomics.

[21] Schneider LS, Dagerman KS, Insel P (2005). Risk of death with atypical antipsychotic drug treatment for dementia - Meta-analysis of randomized placebo-controlled trials. JAMA.

[22] Gareri P, De Fazio P, Manfredi VG, De Sarro G (2014). Use and safety of antipsychotics in behavioral disorders in elderly people with dementia. J Clin Psychopharmacol.

[23] Kales HC, Valenstein M, Kim HM, McCarthy JF, Ganoczy D, Cunningham F, Blow FC (2007). Mortality risk in patients with dementia treated with antipsychotics versus other psychiatric medications. Am J Psychiatr.

[24] Kales HC, Kim HM, Zivin K, Valenstein M, Seyfried LS, Chiang C, Cunningham F, Schneider LS, Blow FC (2012). Risk of mortality among individual antipsychotics in patients with dementia. Am J Psychiatry.

[25] Sultana J, Chang CK, Hayes RD, Broadbent M, Stewart R, Corbett A, Ballard C (2014). Associations between risk of mortality and atypical antipsychotic use in vascular dementia: a clinical cohort study. Int J Geriatr Psychiatry.

[26] Maust DT, Kim HM, Seyfried LS, Chiang C, Kavanagh J, Schneider LS, Kales HC (2015). Antipsychotics, Other Psychotropics, and the Risk of Death in Patients With Dementia Number Needed to Harm. JAMA Psychiatry.

[27] Ballard C, Hanney ML, Theodoulou M, Douglas S, McShane R, Kossakowski K, Gill R, Juszczak E, Yu LM, Jacoby R (2009). Dart-Ad Investigators. The dementia antipsychotic withdrawal trial (DART-AD): long-term follow-up of a randomised placebo-controlled trial. Lancet Neurol.

[28] Danielsson B, Collin J, Jonasdottir Bergman G, Borg N, Salmi P, Fastbom J. Antidepressants and antipsychotics classified with Torsades de Pointes arrhythmia risk and mortality in older adults - a Swedish nationwide study. Br J Clin Pharmacol. 2016;81(4):773-83. 10.1111/bcp.12829 PMC4799929 26574175

[29] Ryan J, Carriere I, Ritchie K, Stewart R, Toulemonde G, Dartigues JF, Tzourio C, Ancelin ML (2008). Late-life depression and mortality: influence of gender and antidepressant use. Br J Psychiatry.

[30] Bingefors K, Isacson D, Knorring LV, Smedby B, Wicknertz K (1996). Antidepressant-treated patients in ambulatory care. Mortality during a nine-year period after first treatment. Br J Psychiatry.

[31] Vinkers DJ, Gussekloo J, van der Mast RC, Zitman FG, Westendorp RG (2003). Benzodiazepine use and risk of mortality in individuals aged 85 years or older. JAMA.

[32] Gisev N, Hartikainen S, Chen TF, Korhonen M, Bell JS (2011). Mortality associated with benzodiazepines and benzodiazepine-related drugs among community-dwelling older people in Finland: a population-based retrospective cohort study. Can J Psychiatry.

[33] Pinot J, Herr M, Robine JM, Aegerter P, Arvieu JJ, Ankri J (2015). Does the Prescription of Anxiolytic and Hypnotic Drugs Increase Mortality in Older Adults?. J Am Geriatr Soc.

[34] Parsaik AK, Mascarenhas SS, Khosh-Chashm D, Hashmi A, John V, Okusaga O, Singh B. Mortality associated with anxiolytic and hypnotic drugs-A systematic review and meta-analysis. Aust N Z J Psychiatry. 2016;50(6):520-33. 10.1177/0004867415616695 26590022

[35] Jennum P, Baandrup L, Ibsen R, Kjellberg J. Increased all-cause mortality with use of psychotropic medication in dementia patients and controls: A population-based register study. Eur Neuropsychopharmacol. 2015;25(11):1906-13. 10.1016/j.euroneuro.2015.08.014 26342397

[36] von Heideken Wågert P, Gustavsson JM, Lundin-Olsson L, Kallin K, Nygren B, Lundman B, Norberg A, Gustafson Y (2006). Health status in the oldest old. Age and sex differences in the Umeå 85+ Study. Aging Clin Exp Res.

[37] Toots A, Littbrand H, Lindelöf N, Wiklund R, Holmberg H, Nordström P, Lundin-Olsson L, Gustafson Y, Rosendahl E (2016). Effects of a High-Intensity Functional Exercise Program on Dependence in Activities of Daily Living and Balance in Older Adults with Dementia. J Am Geriatr Soc.

[38] Rosendahl E, Lindelöf N, Littbrand H, Yifter-Lindgren E, Lundin-Olsson L, Haglin L, Gustafson Y, Nyberg L (2006). High-intensity functional exercise program and protein-enriched energy supplement for older persons dependent in activities of daily living: a randomised controlled trial. Aust J Physiother.

[39] Conradsson M, Lundin-Olsson L, Lindelöf N, Littbrand H, Malmqvist L, Gustafson Y, Rosendahl E (2007). Berg balance scale: intrarater test-retest reliability among older people dependent in activities of daily living and living in residential care facilities. Phys Ther.

[40] Folstein MF, Folstein SE, McHugh PR (1975). “Mini-mental state”. A practical method for grading the cognitive state of patients for the clinician. J Psychiatr Res.

[41] Sheikh J, Yesavage J (1986). Geriatric Depression Scale (GDS): Recent evidence and development of a shorter version. Clin Gerontol.

[42] Lawton MP (1975). The Philadelphia Geriatric Center Morale Scale: a revision. J Gerontol.

[43] Guigoz Y, Vellas B (1999). The Mini Nutritional Assessment (MNA) for grading the nutritional state of elderly patients: presentation of the MNA, history and validation. Nestle Nutr Workshop Ser.

[44] Collin C, Wade DT, Davies S, Horne V (1988). The Barthel ADL Index: a reliability study. Int Disabil Stud.

[45] World Health Organization. International statistical classification of diseases and related health problems. 10th revision. ed. Geneva: World Health Organization; 1992.

[46] American Psychiatric Association., American Psychiatric Association. Task Force on DSM-IV. Diagnostic and statistical manual of mental disorders : DSM-IV-TR. 4th ed. Washington, DC: American Psychiatric Association; 2000. xxxvii, 943 p. p.

[47] van Buuren S (2012). Flexible imputation of missing data.

[48] Drici MD, Clement N (2001). Is gender a risk factor for adverse drug reactions? The example of drug-induced long QT syndrome. Drug Saf.

[49] CredibleMeds Worldwide. CredibleMeds - QTDrugs Lists 2015. Available from: https://www.crediblemeds.org/new-drug-list/. Accessed 15 Jan 2016.

[50] Luyster FS, Buysse DJ, Strollo PJ (2010). Comorbid insomnia and obstructive sleep apnea: challenges for clinical practice and research. J Clin Sleep Med.

[51] Redline S, Yenokyan G, Gottlieb DJ (2010). Obstructive sleep apnea-hypopnea and incident stroke: the sleep heart health study. Am J Respir Crit Care Med.

[52] Gustafsson M, Karlsson S, Lövheim H (2013). Inappropriate long-term use of antipsychotic drugs is common among people with dementia living in specialized care units. BMC Pharmacol Toxicol.

