# Supplementary material for: Psychotropic drug use and mortality in old people with dementia: investigating sex differences
Source: BMC Pharmacol Toxicol. 2017 May 25;18:36. doi: 10.1186/s40360-017-0142-9 (PMC5445267; doi:10.1186/s40360-017-0142-9)
Supplement: Additional file 1: — Statistical procedures. (DOCX 90 kb) [file 40360_2017_142_MOESM1_ESM.docx]

**Appendix 1 – Statistical procedures**

**Variables tested for eligibility as confounders in all Cox regression model**

*Medical conditions and diagnoses:*

Stroke, ever

Alzheimer's disease

Vascular dementia

Other dementia

Non-specified dementia

Depression

Delirium

Psychotic disease

Malignant disease, current

Malignant disease, earlier

Chronic lung disease

Osteoporosis

Diabetes mellitus

Hypertension

Atrial fibrillation

Pacemaker

Other arrhythmia

Angina pectoris

Congestive heart failure

Myocardial infarction, last year.

Myocardial infarction, further back than one year ago

Sleeping disorder

Hip fracture, ever

*Prescribed drugs:*

Digoxin

Diuretics

Calcium blockers

Beta-blockers (oral)

Agents acting on RAAS

Organic nitrates

Analgesics

Opioids

NSAID

ASA

Warfarin

Antibiotics

Antiepileptics

Antidepressants

Neuroleptics

Benzodiazepines

Cholinesterase inhibitors

Ebixa

Number of prescribed drugs

*Scales and measurements:*

Barthel’s ADL index

BMI

GDS-15

MMSE

MNA

PGCMS

**Abbreviations**

ADL= Activities of Daily Living

ASA = Acetylsalicylic Acid

BMI = Body Mass Index

GDS-15 = Geriatric Depression Scale, 15-item version

MMSE = Mini Mental State Examination

MNA = Mini Nutritional Assessment

NSAID = Non Steroidal Anti-inflammatory Drugs

PGCMS = Philadelphia Geriatric Centre Morale Scale

RAAS = renin-angiotensin-aldosterone-system

**Variables qualified for the fully adjusted Cox regression models**

| **Characteristic** | **Antipsychotics** | | | **Antidepressants** | | | **Benzodiazepines** | | |
| --- | --- | --- | --- | --- | --- | --- | --- | --- | --- |
|  | **All** | **Male** | **Female** | **All** | **Male** | **Female** | **All** | **Male** | **Female** |
| Age | X | X | X | X | X | X | X | X | X |
| Sex | X |  |  | X |  |  | X |  |  |
| **Medical conditions** |  |  |  |  |  |  |  |  |  |
| Alzheimer's disease |  |  |  |  | X |  | X |  | X |
| Vascular dementia |  |  |  |  | X |  |  |  |  |
| Delirium, in the last month | X | X | X | X | X | X | X |  | X |
| Depressive disorder |  |  |  |  |  |  |  | (X) |  |
| Angina Pectoris |  |  |  |  |  |  | X | X |  |
| Congestive heart failure |  |  |  |  |  |  | X |  | X |
| Diabetes mellitus |  |  |  |  | X |  |  |  |  |
| Myocardial infarction | X |  | X |  |  |  |  |  |  |
| Stroke, ever |  |  |  | X |  |  |  |  |  |
| Chronic lung disease | X |  | X |  |  | X | X |  | X |
| Sleeping disorder |  | X |  |  | X |  |  | X |  |
| **Prescribed drugs** |  |  |  |  |  |  |  |  |  |
| Antipsychotics | X | X | X |  |  |  |  |  |  |
| Antidepressants |  |  |  | X | X | X |  |  |  |
| Benzodiazepines | X | X |  | X | X |  | X | X | X |
| Analgesics |  |  |  | X | X | X | X | (X) | X |
| Cholinesterase inhibitors |  |  | X | X |  | X |  |  |  |
| Organic nitrates | X |  |  |  | X |  |  |  | X |
| Antibiotics |  | X |  | X | X |  | X | (X) |  |
| ASA |  |  | X |  |  |  |  |  |  |
| Digoxin |  |  |  | X |  | X |  |  |  |
| Agents acting on RAAS |  |  |  | X |  |  |  |  |  |
| Diuretics |  |  |  |  |  |  | X | X | X |
| Number of prescribed drugs | X | X | X | X | X | X | X | (X) | X |
| **Scales and measurements** | |  |  |  |  |  |  |  |  |
| Barthel ADL index | X | X | X | X | X |  | X | X | X |
| BMI |  |  |  | X | X | X | X |  |  |
| GDS-15 | X | X |  | X | X | X | X | X* |  |
| MMSE | X |  | X |  |  |  |  |  |  |
| Included variables (n) | 12 | 9 | 10 | 15 | 15 | 10 | 15 | 7(11) | 11 |

Notes: ASA = acetylsalicylic acid; RAAS = renin-angiotensin-aldosterone-system; ADL = Activities of Daily Living;

BMI = body mass index; GDS = 15-item Geriatric Depression Scale; MMSE = Mini Mental State Examination.

Variables qualified for the adjusted analyses were associated (*P* < 0.15) with mortality and use of respective drug in the total sample and in subgroups of men and women, respectively. Adjustment for age and sex were included in all models. GDS was included in the models with antidepressant use without any required association with mortality or drug use. Variables marked as (X) were included in the model but were removed in a backwards selection process due to a low number of events in relation to the number of included variables. *GDS was removed in a backwards selection process from the model analysing second-year mortality associated with benzodiazepines in men.

**Variables excluded from the fully adjusted Cox regression models**

Some variables were excluded due to singularity or due to multicollinearity (correlations coefficient, r≥0.6). MNA was excluded in all analyses due to singularity caused by the variable “number of drugs”. Similarly, opioids were removed in some analyses due to singularity with analgesics. PGCMS was not included in any analysis due to high correlation (r ≥ 0.6) with GDS-15. GDS-15 was chosen before depressive disorders due to high correlation in some analyses. Angina pectoris was chosen before organic nitrates due to high correlation in some other analyses.

**Sensitivity Analyses**

Since some on the covariates in the multivariate analyses might be considered as possible mediating factors for drug-associated mortality, rather than confounders, cox regression models excluding these varibles have been analysed (Model 3).

*The removed variables are:*

Delirium, in the last month

Angina Pectoris

Congestive heart failure

Diabetes Mellitus

Myocardial infarction

Stroke, ever

Since different covariates were used in the analyses of women and men, as a result of the selection process described in the statistics section, we also performed sex-specific analyses using the covariates selected for the analyses of all participants. The results are presented below as Model 4.

**Cox proportional hazards regression models**

**Model 1 Model 2 Model 3 Model 4**

HR 95% CI HR 95% CI HR 95% CI HR 95% CI

**Antipsychotics (2 year mortality)**

All participants **1.16** 0.94-1.43 **0.91** 0.73-1.14 **0.92** 0.74-1.15

Male participants **1.24** 0.82-1.87 **0.79** 0.51-1.24 **0.84** 0.54-1.31 **0.83** 0.53-1.30

Female participants **1.15** 0.90-1.46 **0.90** 0.70-1.15 **0.90** 0.70-1.15 **0.95** 0.73-1.23

**Antidepressants (2 year mortality)**

All participants **1.01** 0.84-1.21 **0.96** 0.78-1.17 **0.97** 0.80-1.19

Male participants **0.96** 0.67-1.37 **0.61*** 0.40-0.92 **0.64*** 0.42-0.96 **0.66** 0.44-1.01 22

Female participants **1.03** 0.84-1.28 **1.09** 0.87-1.38 **1.11** 0.88-1.40 **1.09** 0.86-1.38

**Benzodiazepines (first-year mortality)**

All participants **1.38*** 1.08-1.77 **1.13** 0.86-1.47 **1.12** 0.86-1.47

Male participants  **2.07*** 1.29-3.32 **1.37** 0.77-2.45 **1.42** 0.80-2.51 **1.67** 0.99-2.81

Female participants **1.21** 0.91-1.62 **0.96** 0.71-1.31 **0.96** 0.71-1.31 **0.96** 0.70-1.31

**Benzodiazepines (second-year mortality)**

All participants **0.95** 0.73-1.24 **0.72*** 0.54-0.96 **0.72*** 0.54-0.96

Male participants **0.90** 0.51-1.59 **0.81** 0.45-1.45 **0.84** 0.47-1.50 **0.74** 0.40-1.39

Female participants **0.98** 0.72-1.33 **0.73** 0.53-1.02 **0.73** 0.52-1.01 **0.72** 0.52-1.00

Notes: Analyses of antipsychotics and benzodiazepines include related drugs, as described in methods.

Hazard ratios marked with * are statistically significant (p<0.05). HR=Hazard ratio. CI=Confidence interval.

Model 1 shows the unadjusted, univariate associations between drug use and mortality.

Model 2 includes all available confounders, selected through the process described in the statistics section.

Interaction Term shows the p-values for the respective interaction terms applied to Model 2.

Model 3 is based on Model 2, but with suspected mediating variables removed.

Model 4 shows the results of sex-specific analyses using the confounders selected for all participants.

**Variables with missing values completed through multiple imputation:**

Barthel ADL index (6.5% missing)

BMI (14.9% missing)

GDS-15 (35.1% missing)

MMSE (14.2% missing)

Myocardial infarction, further back than one year ago (12.2% missing)

**Variables used as predictors in the multiple imputation of missing values:**

*Variables to be imputed:*

Barthel ADL index

BMI

GDS-15

MMSE

Myocardial infarction, further back than one year ago

*Outcomes and predictors:*

Death within two years

Time to death

Antidepressants

Antipsychotics

Benzodiazepines

*Confounders in the Cox regression models:*

Sex

Age

Stroke, ever

Alzheimer's disease

Vascular dementia

Depression

Delirium, last month

Diabetes mellitus

Chronic lung disease

Sleeping disorder

Angina pectoris

Congestive heart failure

Digoxin

Diuretics

Agents acting on RAAS

Organic nitrates

Analgesics

Opioids

ASA

Antibiotics

Cholinesterase inhibitors

Number of prescribed drugs

*Variables correlating with imputed variables or with missing values in the same variables:*

Living in an apartment

Living in a house

Living in a nursing home

Owning your own house or apartment

Good eyesight

Self-rated health

Myocardial infarction, last year

Betablockers, oral

Drugs for constipation

MNA

PGCMS
